# An uncommon case of complete AV block

**DOI:** 10.1186/s12872-022-02866-5

**Published:** 2022-09-29

**Authors:** Federica Valente, Lionel Rozen, Stéphane Carlier, Pascal Godart

**Affiliations:** Department of Cardiology, University Hospital Center Ambroise Paré, Mons, 7000 Mons, Belgium

**Keywords:** Granulomatosis with polyangiitis, Complete atrioventricular block, Cardiac involvement, Multisystem disorder, Pacemaker

## Abstract

**Background:**

Granulomatosis with polyangiitis (GPA) is a rare systemic inflammatory disorder characterized by vasculitis of the small vessels, as well as necrotizing granulomatous lesions, affecting mainly upper and lower respiratory tracts, lungs and kidneys. Cardiac involvement has traditionally been a rare manifestation of GPA, with misleading clinical presentation until late stages. Cardiac conducting tissue involvement is a rare and potentially life-threatening complication.

**Case presentation:**

We report the case of a 45-year-old man diagnosed with GPA with typical symptoms, but also complete atrioventricular (AV) block at the onset of the disease. The echocardiogram was unremarkable but the cardiac magnetic resonance (CMR) showed evidence of inflammation of the basal and septal ventricle walls. Despite effective immunosuppressive therapy, a permanent pacemaker was required for recurring complete AV block.

**Discussion:**

Conduction system abnormalities are a rare manifestation of GPA, due to granulomatous lesions within the conduction system, or arteritis of the atrioventricular nodal artery. Patients are often asymptomatic, so careful and regular screening for cardiac involvement in this multi-system condition is required, often with echocardiogram, electrocardiogram (ECG) monitoring and CMR. Early immunosuppressive treatment may reverse a complete AV block but a pacemaker implantation may sometimes be necessary.

## Introduction

Granulomatosis with polyangiitis (GPA, also known as Wegener’s granulomatosis) is a rare systemic inflammatory disorder of unknown etiology that is characterized by necrotizing vasculitis combining inflammation of the vascular wall of small vessels and granulomatosis, peri- and extravascular [1, 2].

Clinically, it is characterized in its complete form by ENT signs, lung damage and kidney damage [3].

Cardiac involvement is a relatively rare manifestation of GPA, affecting 6–25% [4, 5] of patients with this disorder. The most common cardiac manifestations of GPA are pericarditis, myocarditis, coronary arteritis and valvular lesions 6. Cardiac conducting tissue defects, including complete heart block may occur.

Here we report a case of complete atrioventricular block in a patient with GPA and how this was managed.

## Timeline

**Table:** 

Time	Events
July 2021	Recurrent episodes of sinusitis, not responsive to antibiotics
30 July 2021	Patient admitted to hospital for ear pain and loss of hearing, nasal crusting and fever
30 July 2021	Biology: leukocytosis, high protein C reactive Rhinoscopy: crusty and inflammatory mucous membrane; systemic disease?
2 August 2021	c-ANCA anti-PR3 positive
2 August 2021	Thoracic-CT : lung nodules and sub-glottic tracheal stenosis
3 August 2021	Patient presented pre-syncopal episode
3 August 2021	ECG showed sinus tachycardia Transthoracic echocardiogram was normal.
3 august 2021	Treatment initiated with pulses of methylprednisolone (500 mg twice/day)
4 August 2021	24 Holter ECG showed AV block 2nd and 3rd degree
5 August 2021	Cardiac magnetic resonance imaging showed myocardial edema in septal wall
5 August 2021	Treatment with Rituximab (375 mg/m^2^)
6–11 August 2021	Recurrent complete AV block
10 August 2021	Treatment with Rituximab (375 mg/m^2^) 2nd dose
11 August 2021	Permanent pacemaker implantation
18–25 August 2021	Treatment with Rituximab
29 September 2021	Pacemaker analysis: sinus rhythm, pacing rate < 1%

## Case presentation

A 45-year-old man was admitted to our hospital with a history of ear pain and loss of hearing, nasal crusting, exertional dyspnea and fever for a month. He was a factory worker, smoker and known for hypertension treated by calcium antagonist and ACE inhibitor. He presented in the past few months multiples episodes of nasal congestion treated as chronic sinusitis with antibiotics and inhaled corticosteroids without improvement.

On physical examination, his temperature was 37.5 °C, oxygen saturation 97%, blood pressure 120/60 mmhg, pulse 102/min and regular. The nasal exam showed right nasal septal deviation and crusty and inflammatory mucous membrane bleeding at the contact. Papular skin lesions were present on the elbows. Pulmonary and cardiac auscultation was normal; no organomegaly nor signs of synovitis was present.

Laboratory evaluation showed normocytic normochromic anemia (10 g/dl) with increased ferritin and low serum iron, transferrin and transferrin saturation, typical of anemia of inflammation, leukocytosis (13,000/mL, neutrophiles 73%) and high-sensitivity C-reactive protein (158 mg/dl). Liver and kidney function tests were normal, with mild hematuria and proteinuria (200 mg/24 h). There was no nutrient deficiency, no signs of hemolysis, no electrolytes disorders and normal thyroid tests.

The thoracic computerized tomography (CT) scan showed multiple lung nodules and sub-glottic tracheal stenosis.

Biopsies of the nasal cavity and skin lesions were performed.

Further laboratory evaluation by indirect immunofluorescence showed a characteristic cANCA (cytoplasmic antineutrophil cytoplasmic antibodies) pattern. The patient’s serum was also positive for antibodies to proteinase-3 (anti-PR3) by enzyme-linked immunosorbent assay (ELISA).

**Fig. 1:**
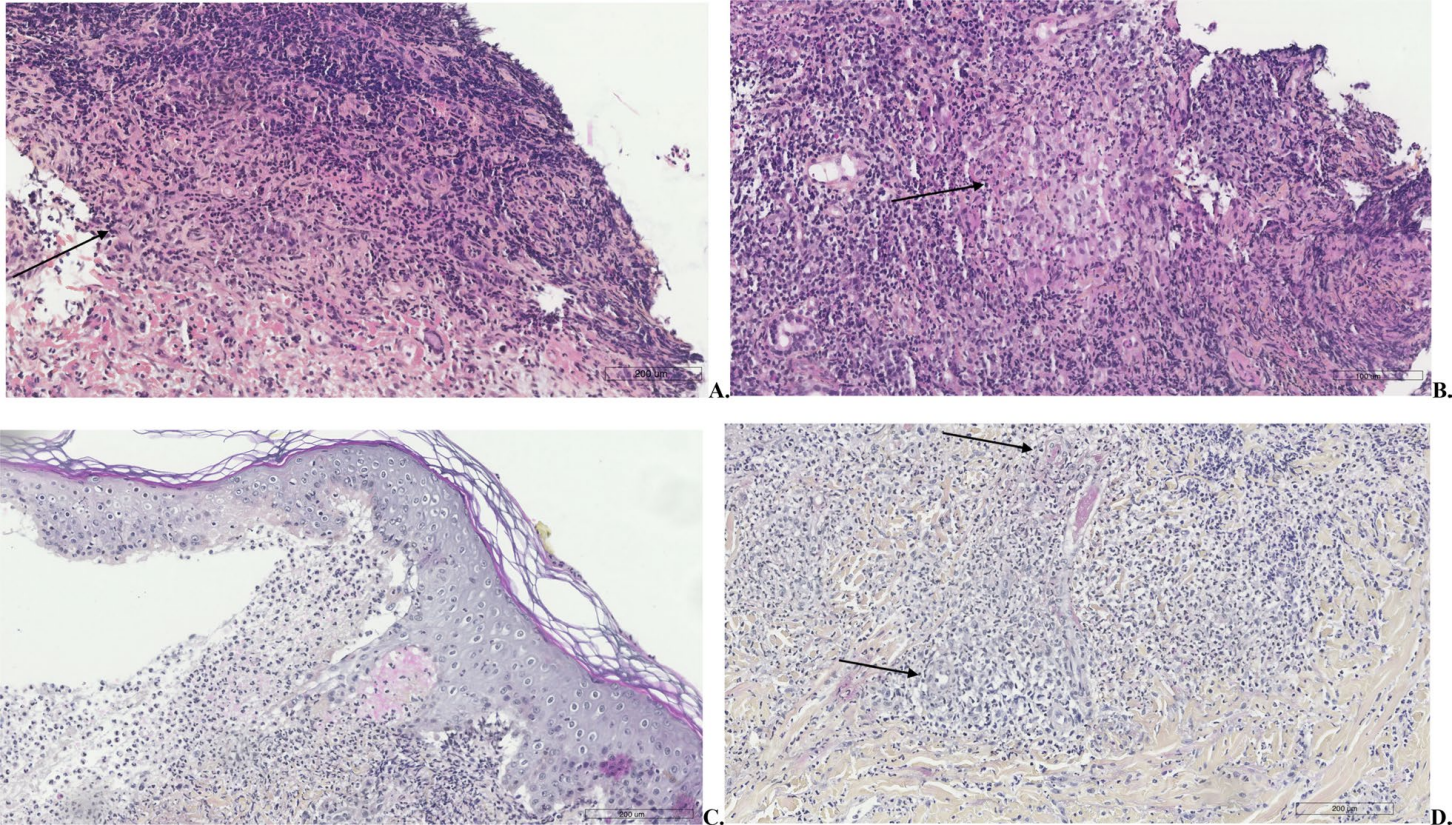
**a, b** Nasal granulomatous. Presence in nasal biopsies of an inflammatory granulation tissue, formed of histiocyte lymphocytes, of a few rare plasmacytes and especially many granulocytes. There is no caseous necrosis. Presence of a small granuloma with giant cells embedded in the inflammatory reaction (arrow) (H.E. x 20 in **a**, x 40 in **b**). **c** Skin bleb. Subepidermal detachment with formation of a space partially filled with inflammatory and fibrinoid material (H.E. x 20). **d** Skin granulomatous. Histologic section of skin biopsy showing signs of vasculitis (arrow) with parietal necrosis fibrinoid (H.E. x 20)

The diagnosis of Granulomatosis with Polyangiitis was made according to the ACR/EULAR 2017 Classification Criteria, based on the typical clinical (bloody nasal discharge and ulcers, hearing reduction) and biological criteria (cANCA and antiPR3), confirmed by classic histological lesions of granuloma formation and necrotizing vasculitis found in the nasal and skin biopsy (Fig. 1).

On the 3rd day following admission to hospital, the patient experienced a pre-syncopal episode, with no loss of consciousness, no chest pain and no palpitations.

The electrocardiogram documented sinus tachycardia (110 bpm) and supra ventricular extrasystoles.

The transthoracic echocardiography showed a normal ejection fraction, with no regional wall motion abnormalities, no valvular disease and no pericardial effusion. The diastolic function was normal with a low E/e’ ratio < 14, a not dilated left atrium (volume 25 ml/m2) and a peak TR velocity < 2,8 m/s; no intracardiac mass were viewed.

The levels of myocardial necrosis markers such as troponin and N-terminal pro brain natriuretic peptide were normal.

**Fig. 2:**
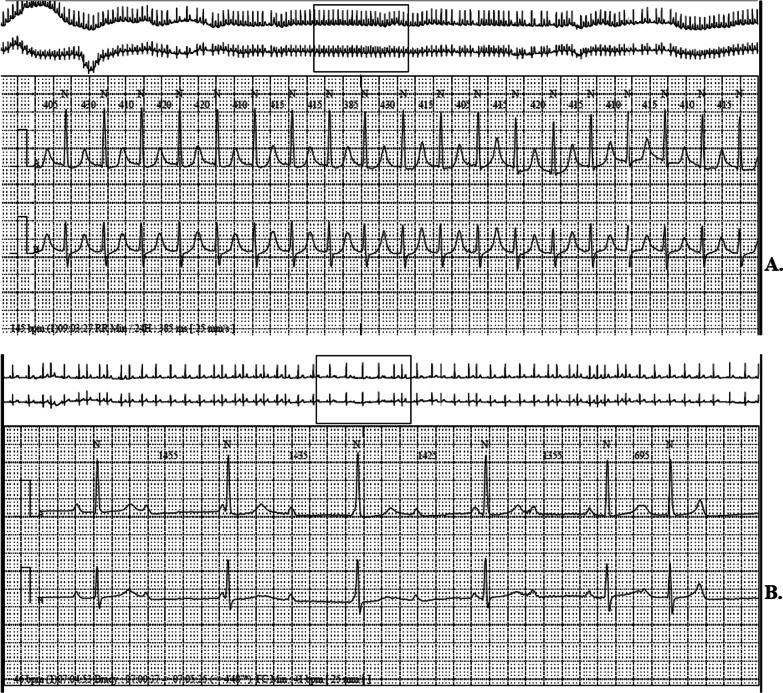
24-hour Holter ECG. **a** Atrial tachycardia. **b** 3rd degree AV block

The 24-h Holter ECG was highly pathological showing profuse supraventricular extrasystoles (25,000/24 h) with atrial tachycardia bursts at about 150/min, and second- and third-degree atrioventricular block diurnal and nocturnal (Fig. 2).

**Fig. 3:**
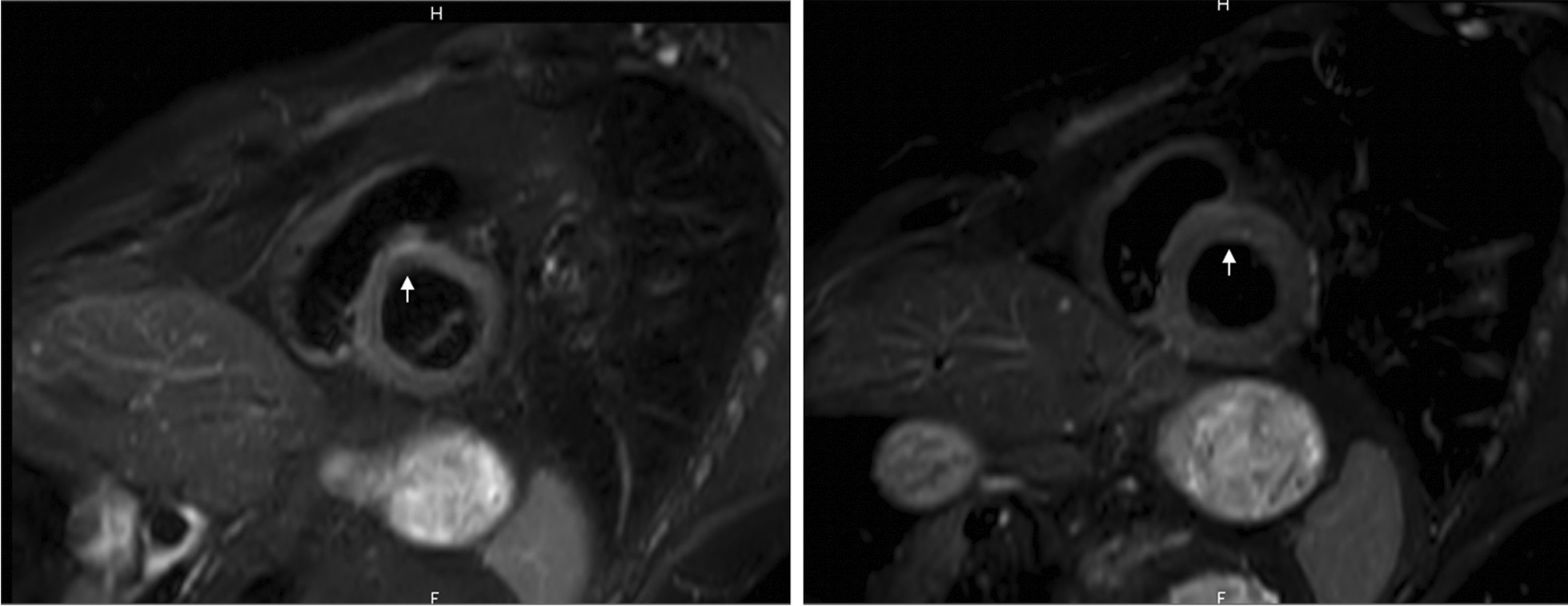
Cardiac magnetic resonance imaging. Cardiac magnetic resonance imaging showing hypersignal in T2 weighted sequences in septo-basal wall (arrow)

The cardiac magnetic resonance showed small foci of increased intramyocardial sign on T2 weighted sequences in the septal and lateral walls, suggestive of myocardial edema, possibly related to the GPA (Fig. 3).

After the exclusion of other causes of AV block, such as ischemic disease, metabolic or drug related causes, infectious disease (Lyme, tuberculosis, syphilis) or infiltrative myocardial disease, we considered the AV block as part of cardiac involvement of the GPA.

A treatment with pulses of methylprednisolone (500 mg twice/day) and Rituximab (375 mg/m2) at one dose every week for four weeks was started, according to the 2021 American College of Rheumatology/Vasculitis Foundation Guideline for the treatment of active severe GPA [7].

Despite steroid treatment for 10 days and two doses of Rituximab, the patient continued to experience symptomatic high-grade AV block with prolonged episodes of supraventricular tachycardia.

In agreement with infectiologists and internists, given the persistence of brady and tachyarrhythmia symptoms despite treatment, and given the higher risk of infection of a temporary pacing, we decided to implant a dual chamber pacemaker.

With continuing immunosuppressive therapy for four weeks, the upper respiratory symptoms, as well as general and cardiac symptoms, improved significantly. The inflammatory markers levels normalized (CRP 9 mg/L).

In the follow-up one month later, we could start beta-blockers, his ECG returned to normal sinus rhythm and pacing rate was less than 1%. In the absence of recurrence of sustained supraventricular arrhythmia, no ablation was proposed. A follow-up CMR is planned in the year.

## Discussion and conclusions

Granulomatosis with polyangiitis is necrotizing vasculitis combining vascular wall inflammation and peri and extravascular granulomatosis [1]. Clinically, GPA is characterized by ENT signs, lung and renal involvement, but any organ system can be affected [1–3].

Cardiac involvement is less common, occurring in 6–25% [3–5] of cases and is secondary to necrotizing vasculitis with granulomatous infiltrates [6–8]. In a retrospective study of 27 patients with cardiac involvement in GPA, Forstot et al. [5] reported the following histopathological findings: 50% pericarditis, 50% coronary arteritis, 25% myocarditis; 21% valvulitis/endocarditis; 17% conduction system granulomata, 13% sinus node and AV node arteritis and 11% myocardial infarction.

Atrial tachycardia, atrial fibrillation and flutter are the most common arrhythmias found in patients with GPA; ventricular arrhythmias are usually noted in hearts with structural damage [5] and conduction defects are less frequently recognized [9].

Complete AV block is a rare and severe manifestation of cardiac involvement in GPA with less than 20 cases reported in the published literature [5–16]. It is usually present at disease onset, often asymptomatic despite active autoimmune disease.

Echocardiographic abnormalities are found in more than 70% of patients, most commonly valvular disease and pericardial effusion.

Recently, the cardiac magnetic resonance has emerged as a novel technique providing an accurate assessment of myocardial function and structure, including detection of myocardial inflammation [12,15].

Indeed, in our patient, transthoracic echocardiography was considered normal, but CMR imaging revealed presence of myocardial oedema on septal wall, which might explain third AV block.

This suggests that CMR may be more sensitive than transthoracic echocardiography for evaluating GPA-related cardiac involvement. Moreover, it seems to be a reliable tool for monitoring therapeutic efficacy.

Treatment includes corticosteroids, cyclophosphamide, rituximab, temporary pacing wire, and pacemaker implantation [9]. Early immunosuppressive treatment may reverse a complete AV block and obviate the need for permanent pacemaker implantation. However, the chances and time course of resolution of AV block with immunosuppressive therapy are not clear.

As described in the case report of Cassidy et al. [9], despite the remission of the inflammatory disease with immunosuppressive therapy, the patient continued to experience symptomatic high-grade AV block, requesting pacing support, emphasizing then that GPA remission is not always synonymous of AV conduction recovery.

A recent case of Taskesen et al. [16] described the history of a patient with a large intracardiac mass infiltrating the A-V node, causing a symptomatic complete AV block requiring a pacemaker. In this case, despite regression of the intracardiac mass on immunosuppressive therapy, the complete AV block did not resolve and the patient was pacemaker dependent. Unfortunately, the patient died of septic shock and the cardiac autopsy could show transmural fibrosis and chronic inflammation of interatrial septum.

In our patient, the decision to proceed with permanent pacemaker implantation was based on the presence of disabling supraventricular arrhythmia and symptomatic 2nd and 3rd degree AV block, the necessity of betablockers treatment and the persistence of arrythmia during the immunosuppressive treatment.

We didn’t choose a temporary pacing system due to the much higher risk of infections that would have been increased by the immunosuppressive treatment. As indicated in the large prospective study of Klug et al., the patients in whom a temporary pacing system was present at the time of implantation of the permanent antiarrhythmic systems were more than twice as likely to develop device-related infections as patients who were not temporarily paced [17].

In conclusion, this case report describes a patient with persistent AV block in granulomatosis with polyangiitis, in the current era of modern treatment like Rituximab as well as innovative diagnosis tool as CMR.

All patients diagnosed with GPA should be screened with a baseline electrocardiogram and a transthoracic echocardiogram to document cardiac involvement and alert clinicians to those at risk of further cardiac complications. In this screening indication, the place of CMR is promising by allowing earlier detection than echocardiography of inflammatory and edematous lesions and their evolution.

Reversibility of conduction disorder with immunosuppressive therapy is unpredictable and permanent pacing should be considered.

Given the frequencies of high degree conduction disorder, a regular follow-up by ECG and Holter is strongly advised.

## Data Availability

All data generated or analyzed during this study are included in this published article.

## Abbreviations

ACE: Angiotensin-converting enzyme
AVB: Atrioventricular block
CMR: Cardiac magnetic resonance
GPA: Granulomatosis with polyangiitis
TR: Tricuspid regurgitation
